# Arbuscular Mycorrhizal Fungi Enhance Antioxidant Defense Systems in Sugarcane Under Soil Cadmium Stress

**DOI:** 10.3390/plants14182916

**Published:** 2025-09-19

**Authors:** Gloria Magaly Paladines-Beltrán, Nathalia Alejandra Venegas, Juan Carlos Suárez

**Affiliations:** 1 Programa de Doctorado Ciencias Naturales y Desarrollo, Facultad de Ciencias Agropecuarias, Universidad de la Amazonia, Florencia 180001, Colombia; 2 Centro de Investigaciones Amazónicas CIMAZ Macagual César Augusto Estrada González, Grupo de Investigación en Productos Naturales Amazónicos GIPRONAZ, Universidad de la Amazonia, Florencia 180001, Colombia; n.venegas@udla.edu.co; 3 Programa de Ingeniería Agroecológica, Facultad de Ingeniería, Universidad de la Amazonia, Florencia 180001, Colombia; ju.suarez@udla.edu.co; 4 Centro de Investigaciones Amazónicas CIMAZ Macagual César Augusto Estrada González, Grupo de Investigaciones Agroecosistemas y Conservación en Bosques Amazónicos-GAIA, Florencia 180001, Colombia

**Keywords:** oxidative stress, bioremediation, antioxidant enzymes, photosynthetic pigments, fungal colonization, metallic tolerance

## Abstract

Cadmium (Cd) is a toxic metal that affects living organisms even at low concentrations, causing physiological alterations and biomass reduction in plants. Arbuscular mycorrhizal fungi (AMF) represent a biological strategy that increases tolerance to heavy metals, although their specific mechanisms in sugarcane remain poorly understood. To address this knowledge gap, an open-field experiment was conducted to evaluate the effects of AMF on Cd accumulation, oxidative stress, photosynthetic pigments, enzymatic antioxidant system, and non-enzymatic antioxidant compounds in sugarcane variety CC 01-1940, using a randomized block design. Results showed that AMF established symbiosis with plants, retaining Cd in the roots and reducing its translocation to leaves. Additionally, they decreased Cd-induced oxidative stress by reducing lipid peroxidation (MDA) and proline content. Although an initial decrease in photosynthetic capacity was observed, AMF helped maintain stable levels of photosynthetic pigments, preserving photosynthetic efficiency. They also activated antioxidant enzymes and increased antioxidant compounds such as reduced glutathione (GSH), non-protein thiols (NP-SH), ascorbic acid (AA), and phytochelatins (PC). These findings demonstrate that symbiosis with AMF protects sugarcane plants from cellular oxidative damage and reduces Cd concentrations in leaves. Therefore, the use of AMF represents an effective strategy to improve the antioxidant defense and resistance of sugarcane plants to cadmium stress.

## 1. Introduction

Sugarcane (*Saccharum officinarum*) is a crop of high economic value, particularly in tropical and subtropical regions characterized by acidic soils [1]. Its importance lies in its multiple industrial, economic, medicinal, and food applications, on which a significant portion of the world’s population depends. For the year 2023, South America recorded a production exceeding 867 million tons, with Colombia contributing 32.4 million tons [2]. This energy crop provides 70% of the global sugar supply, in addition to generating raw materials for various industries through its byproducts. However, its extensive cultivation has raised concerns regarding potential contamination of the human food chain. Studies have shown that sugarcane can accumulate considerable amounts of cadmium (Cd) in its tissues [3,4], tolerating concentrations of up to 500 μM Cd^2+^ in solution, with an accumulation of 451 mg kg^−1^ dry weight in shoots [5,6].

Cadmium shows increased solubility and mobility in acidic soils, facilitating its transport from root to stem and leaves in the soil-plant system, which increases its incorporation into the food chain [7]. This element is a highly toxic contaminant that, even in trace concentrations, significantly affects plants, animals, and other living organisms [8]. In humans, it can trigger various types of cancer, affect the skeletal, respiratory, renal, and reproductive systems, as well as cause cardiovascular diseases [9,10]. In plants, Cd interferes with essential metabolic processes, altering growth and development, causing significant physiological, ultramorphological, and biochemical anomalies, resulting in a substantial reduction in biomass production [11,12,13].

The Cd phytotoxicity induces oxidative damage through the overproduction of reactive oxygen species (ROS) in the cytosol, a process initiated by free metal ions [14]. This damage affects multiple cellular components, including mitochondrial proteins, lipid membranes, and DNA, while also inhibiting antioxidant enzymes, which can lead to cell death [15,16]. As a defense mechanism, plants have developed both enzymatic and non-enzymatic antioxidant systems to regulate and reduce excessive ROS levels [16,17]. The antioxidant systems include Enzymatic: Catalase (CAT, E.C.1.11.1.6), Ascorbate Peroxidase (APX, E.C. 11.1.11.1), Glutathione Reductase (GR, EC 1.6.4.2), Superoxide Dismutase (SOD, E.C.1.15.1.1), Peroxidase (POD, E.C.1.11.1.7), Non-enzymatic: Reduced Glutathione (GSH), Total Non-protein Thiols (NP-SH). The latter strengthens the defensive system through the activation of phytochelatins via phytochelatin synthase [13,16].

Plants have developed sophisticated mechanisms for heavy metal detoxification, among which the formation of complexes with phytochelatins (PC) stands out [18]. These PCs respond to the presence of toxic metals by forming PC-metal and PC-metalloid complexes, characterized by their extraordinary stability due to their high cysteine and glutathione content. These complexes, synthesized through an enzymatic reaction catalyzed by PC synthase (PCS), are subsequently stored in cellular vacuoles, where the harmful impact of Cd is significantly reduced [19]. In this context, arbuscular mycorrhizal fungi (AMF) emerge as natural enhancers of these defense mechanisms. The AMF not only increases the exploration area of the root system and improves metal ion solubility through chelator production [20,21,22] but also promotes PC formation and activation of the antioxidant system. This capability positions them as a promising biological solution for increasing plant resistance to stress from toxic metals such as Cd [23].

The benefits of AMF extend beyond protection against heavy metals. These organisms establish a “living bridge” between soil and plant [24], facilitating bidirectional nutrient translocation: from soil to roots and carbon transport in the opposite direction. This symbiosis results in improved growth, overall nutritional status of the plant [25], phytohormone level, photosynthetic activity, real quantum yield, and heat dissipation level [26,27,28]. Considering this background, it is fundamental to investigate the potential of the symbiosis between AMF and sugarcane to strengthen defense mechanisms against Cd stress. This study aims to evaluate the protective role of arbuscular mycorrhizal fungi (AMF) in sugarcane plants under cadmium stress conditions by assessing the colonization efficiency and its impact on cadmium translocation patterns, analyzing the modulation of enzymatic and non-enzymatic antioxidant defense mechanisms, determining the effectiveness of AMF symbiosis in mitigating oxidative stress and maintaining photosynthetic capacity, as well as investigating the temporal dynamics of antioxidant responses in AMF-colonized sugarcane exposed to cadmium stress. This research seeks to provide insights into the potential use of AMF as a biological strategy for enhancing sugarcane resilience in cadmium-contaminated soils and developing sustainable phytoremediation approaches. Symbiosis between arbuscular mycorrhizal fungi (AMF) and sugarcane is expected to enhance antioxidant defense mechanisms and increase tolerance to cadmium stress by activating enzymatic and non-enzymatic defense systems.

## 2. Results

### 2.1. Colonization of Arbuscular Mycorrhizal Fungi and Cadmium Content

**Figure 1:**
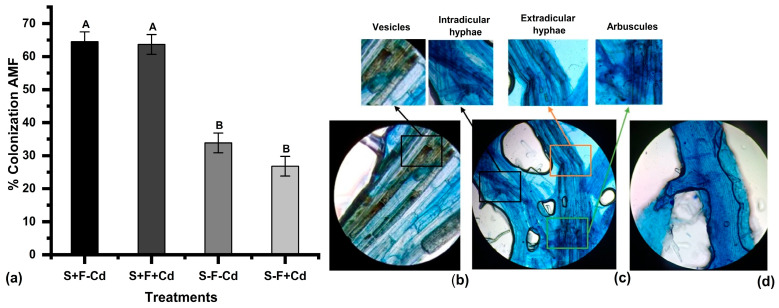
(**a**) Colonization percentage by arbuscular mycorrhizal fungi (AMF) in sugarcane plants exposed to Cd (**b**,**c**) root sample hyphae enriched with AMF, observed at 40×. (**d**) Control root sample without AMF. Different letters indicate significant differences according to the DGC test (*p* < 0.05). Treatments S-F-Cd, S-F+Cd, S+F-Cd, S+F+Cd, (S: soil, F: Fungi; Cd: Cadmium 50 mg kg^−1^; +: Addition; -: Without addition).

Treatments with inoculum demonstrated substantial colonization levels in sugarcane roots, evidenced by the abundant presence of hyphae, vesicles, and arbuscules. This colonization was significantly higher than that observed in native AMF (arbuscular mycorrhizal fungi) from the region’s soil (Figure 1). The S+F-Cd and S+F+Cd treatments achieved colonization rates of 64%, while in treatments without inoculum, colonization was below 35%. These results demonstrate the effectiveness of commercial inoculum in enhancing mycorrhizal symbiosis. Adequate colonization levels were established before the addition of the metal, which was fundamental to ensure the validity of the experimental process.

The Cd concentration was determined in leaves and rhizosphere soil to assess its distribution (Table 1). The results revealed a statistically significant increase in Cd accumulation in both leaves and soil in treatments with CdCl_2_ addition. The soil Cd content in S-F+Cd exceeded the treatment concentration (50 mg kg^−1^), probably due to the initial soil Cd content (0.16 mg Kg^−1^) and the residual from the pretreatment of the experimental soil with phosphate-based fertilizers, which are a known source of Cd [29]. Higher Cd accumulation was observed in leaves from the S-F+Cd treatment compared to S+F+Cd, while the latter showed lower Cd retention in soil. These results demonstrate that Cd underwent translocation between soil and sugarcane leaves in metal-containing treatments, with this translocation being reduced in the presence of stronger colonization by AMF (Table 1).

### 2.2. Analysis of Photosynthetic Pigments and Biochemical Stress Markers

**Figure 2:**
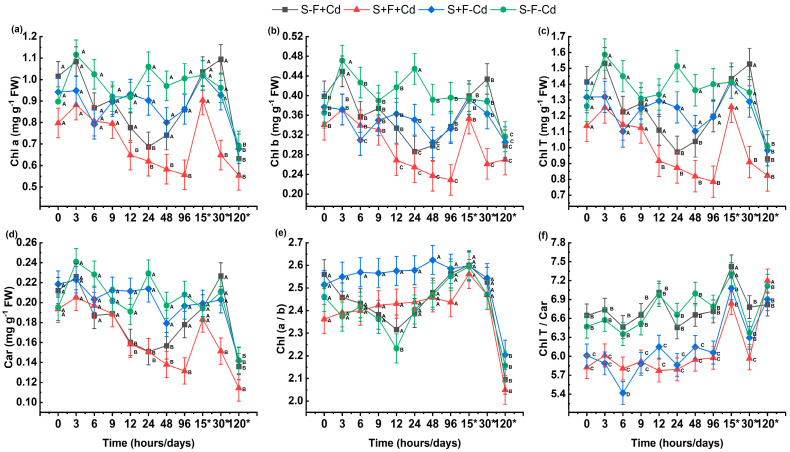
Effect of AMF on photosynthetic pigment content in sugarcane subjected to Cd. (**a**) Chl a: Chlorophyll a; (**b**) Chl b: Chlorophyll b; (**c**) Chl t: Total chlorophyll; (**d**) Car: Carotenoids; (**e**) Chl a/b: Chlorophyll a and b ratio; (**f**) Chl T/car: Total chlorophyll and carotenoids ratio. Data are represented as mean value ± S.E. Different letters indicate significant difference according to the DGC test at *p* < 0.05. Treatments S-F-Cd, SF+Cd, S+F-Cd, S+F+Cd, (S: soil, F: Fungi; Cd: Cadmium 50 mg kg^−1^; +: Addition; -: Without addition); * 15, 30 and 120 correspond to days.

Photosynthetic pigment fractions were significantly affected by Cd and AMF inoculation (Figure 2). In all treatments, the contents of chlorophyll a, b, total chlorophyll, carotenoids, and the ratios of Chl a/b and total Chl/Car decreased significantly from initial hours to 48 h, showing an increase at 15 days. The Chl a/b and total Chl/Car ratios (Figure 2e,f) in inoculated plants showed no statistically significant differences throughout the experimental period up to 120 days, with a decrease observed in the Chl a/b ratio and an increase in total Chl/Car. Cd addition significantly reduced carotenoid content in plants. Treatments with AMF and without metal showed higher carotenoid and chlorophyll b content between 12 and 48 h.

**Figure 3:**
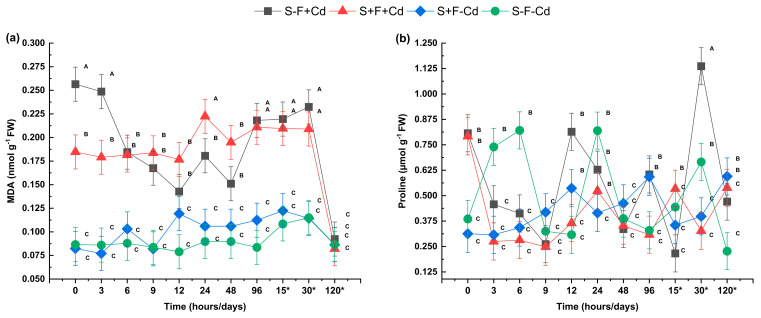
Effect of AMF on oxidative damage indicators and proline in sugarcane subjected to Cd (**a**) MDA: Malondialdehyde (**b**) Proline. Data are represented as mean value ± S.E. Different letters indicate significant difference according to the DGC test at *p* < 0.05. Treatments S-F-Cd, S-F+Cd, S+F-Cd, S+F+Cd, (S: soil, F: Fungi; Cd: Cadmium 50 mg kg^−1^; +: Addition; -: Without addition); * 15, 30 and 120 correspond to days.

Lipid peroxidation, estimated through malondialdehyde (MDA) content, increased significantly in treatments containing Cd (Figure 3a). However, during the initial hours, the S+F+Cd treatment showed significantly lower levels than S-F+Cd. A significant increase in lipid peroxidation was observed at 96 h, 15 days, and 30 days. At 120 days, MDA levels decreased in all treatments without significant differences between them, indicating a reduction in oxidative stress. Proline content, a compatible solute that increases under stress conditions, was used as a stress indicator. It showed an increase in the S-F+Cd treatment at 0, 12, 96 h, and 30 days (Figure 3b). Treatments with AMF inoculum tended to show lower proline values at several time points, suggesting a potential role of AMF in modulating stress responses, although the high variability observed prevents drawing definitive conclusions.

### 2.3. Enzymatic Antioxidant Defense System

**Figure 4:**
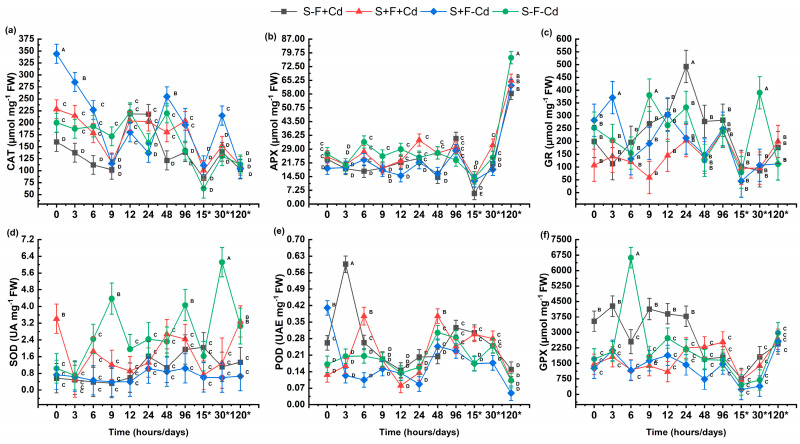
Effect of AMF on enzymatic antioxidants in sugarcane exposed to Cd (**a**) CAT: Catalase; (**b**) APX: Ascorbate peroxidase; (**c**) GR: Glutathione Reductase; (**d**) SOD: Superoxide Dismutase; (**e**) POD: Peroxidase; (**f**) GPX: Guaiacol Peroxidase. Data are represented as mean value ± S.E. Different letters indicate significant difference according to the DGC test at *p* < 0.05. Treatments S-F-Cd, S-F+Cd, S+F-Cd, S+F+Cd, (S: soil, F: Fungi; Cd: Cadmium 50 mg kg^−1^; +: Addition; -: Without addition); * 15, 30 and 120 correspond to days.

The enzymatic antioxidant response in sugarcane leaves is shown in Figure 4. The CAT activity showed a higher response during initial hours in AMF treatments, significantly decreasing to 120 days (Figure 4a). APX enzyme showed inhibition in the initial hours, significantly increasing at 120 days (Figure 4b). The GR enzyme demonstrated a higher active response in the presence of AMF compared to the control; however, the most significant activation was observed at 24 h in plants exposed to cadmium without AMF (Figure 4c). The SOD activity was found active from hour 0 when sugarcane plants were in symbiosis with AMF, but as time progressed, enzyme inhibition occurred relative to the control; additionally, plants with AMF and metal showed activation at 120 days (Figure 4d). The POD enzyme showed an active response in the first hours in treatments with metal and AMF, significantly decreasing at 120 days (Figure 4e). The GPX enzyme showed activity in initial hours up to 24 h in plants exposed to Cd; however, the addition of AMF did not show significant effects during the experimental period (Figure 4f).

### 2.4. Non-Enzymatic Antioxidant Defense System

**Figure 5:**
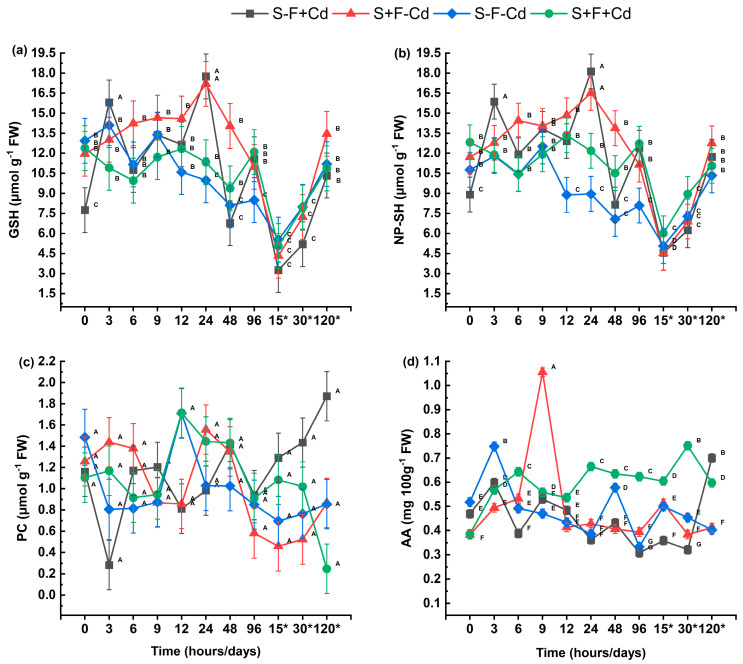
Impact of AMF on non-enzymatic antioxidant compounds in sugarcane exposed to Cd (**a**) GSH: Reduced Glutathione; (**b**) NP-SH: Non-protein thiols; (**c**) PC: Phytochelatins; (**d**) AA: Ascorbic acid. Data are represented as mean value ± S.E. Different letters indicate significant difference according to the DGC test at *p* < 0.05. Treatments S-F-Cd, S-F+Cd, S+F-Cd, S+F+Cd, (S: Soil, F: Fungi; Cd: Cadmium 50 mg kg^−1^; +: Addition; -: Without addition); * 15, 30 and 120 correspond to days.

The non-enzymatic antioxidant compounds in sugarcane leaves are shown in Figure 5. At 3 and 24 h, the highest concentrations of reduced Glutathione (GSH) and non-protein thiols (NP-SH) were observed in the S-F+Cd and S+F-Cd treatments, showing statistically significant differences compared to other treatments (Figure 5a,b). However, at 96 h, a decrease in concentrations was observed, followed by an increase at 120 days. The phytochelatin (PC) content (Figure 5c) throughout the experimental period showed no statistically significant differences between treatments, which may be related to the activation of enzymes that generate plant tolerance. The highest Ascorbic Acid (AA) content (Figure 5d) was observed in the initial hours in plants with AMF (Arbuscular Mycorrhizal Fungi). However, after 12 h, there was a significant decrease in treatments with metal and AMF.

### 2.5. Principal Component Analysis of Photosynthetic Pigments and Antioxidant Systems in AMF-Colonized Sugarcane Under Cd Stress

**Figure 6:**
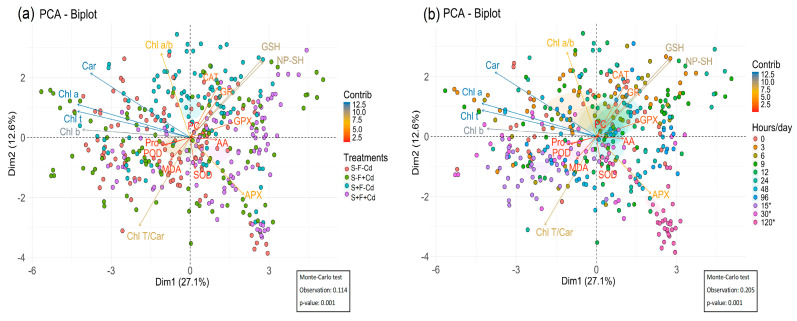
(**a**) Principal components by treatments S-F-Cd, S-F+Cd, S+F-Cd, S+F+Cd, (S: soil, F: Fungi; Cd: Cadmium 50 mg kg^−1^; +: Addition; -: No addition) and (**b**) Principal components by time, * 15, 30, and 120 corresponds to days.

The principal component analysis performed on the matrix of photosynthetic pigments, oxidative damage, enzymatic and non-enzymatic antioxidant systems, considering treatments and experimental time as factors, explained 39.7% of the total data variability (Dim 1: 27.1%; Dim 2: 12.6%). According to the Monte Carlo test, a significant effect of the variables in relation to the factors was found (*p*-value: 0.001). Dim 1 related treatments S+F+Cd, S+F-Cd, and S-F+Cd at 9, 12 h, and 120 days with activation of enzymatic and non-enzymatic antioxidant systems, MDA, and the ratios of Chl a/b and Chl T/Car (Figure 6a,b), while Dim 2 associated treatments S-F-Cd and S-F+Cd at 3, 24 h, and 30 days with proline content, AA, and photosynthetic pigments Chl a, Chl b, total Chl, and carotenoids (Figure 6a,b).

**Figure 7:**
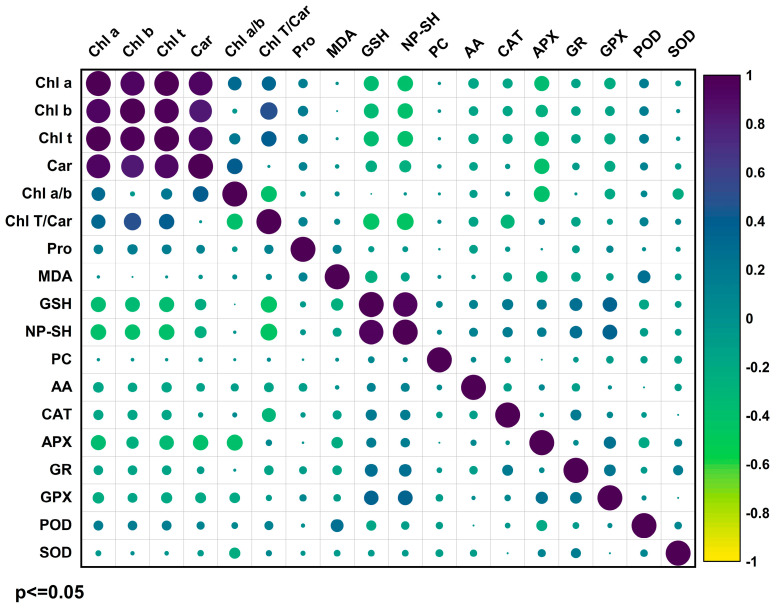
Pearson correlation coefficients between photosynthetic pigments, oxidative damage, the enzymatic antioxidant system, and non-enzymatic antioxidant compounds of sugarcane exposed to Cd with the addition of AMF *p* ≤ 0.05.

The correlations observed between variables in the PCA were confirmed by Pearson’s linear correlation coefficients, shown in Figure 7 with a 95% confidence level. Chlorophyll contents Chl a, b, and Car showed a highly significant linear correlation with Chl T content. GSH positively correlated with NP-SH, while enzymes CAT, APX, GR, GPX, and POD positively correlated with GSH and negatively with Chl T. AA, APX, GSH, NP-SH, and GR negatively correlated with photosynthetic pigments. SOD negatively correlated with the Chl a/b ratio. Additionally, proline contents correlated with Chl a, b, Car, T contents, Chl T/Car ratio, and MDA (Figure 7).

## 3. Discussion

AMF showed a substantial colonization level in sugarcane plants, which allowed the analysis of metal translocation from soil to aerial tissues. This adaptability of AMF is attributed to the edaphoclimatic conditions produced by the mutualistic symbiosis with the plant, which releases exudates and organic compounds such as sugars, organic acids, and amino acids, promoting hyphal morphogenesis with the development of arbuscules and vesicles [30,31]. The symbiosis with plant roots generated extraradical fungal mycelium (EFM) development, helping to intercept and chelate heavy metals by restricting metal movement through hydroxyl and carboxyl groups [32,33,34], contributing to reduced translocation to leaves. This behavior was similar to that reported by Song et al. [35], where 98% of Cd was retained in the soil-root system, while only 2% was translocated to leaves, attributed to the plant’s active response through AMF stimulation of defense mechanisms and adaptability in regulating metal ions through chelation and retention [36].

Cd exposure was associated with increases in lipid peroxidation (MDA) and proline content, although the magnitude of these changes varied over time. AMF tended to reduce MDA and proline levels compared to non-inoculated treatments, suggesting an improvement in plant tolerance to Cd stress through modulation of antioxidant system activity [37]. AMF presence exerted a protective effect by promoting a favorable redox balance, thus improving plant resistance to Cd stress [38]. Similar results were reported by Li et al. [26] in *Medicago truncatula* seedlings with AMF exposed to Cd, where AMF inoculation decreased metal and MDA content in leaf samples while increasing plant biomass. This effect is attributed to the increase in various amino acids and organic acids, which facilitate a metal remediation mechanism by AMF.

The Cd exposure affected photosynthetic efficiency through foliar chlorosis, as observed in the studied plants. Both mycorrhizal and non-mycorrhizal plants showed decreased photosynthetic content due to metal exposure, consistent with findings reported in studies on *Sesbania drummondii* and *Robinia pseudoacacia* L. [39,40]. The Chl a/b and Chl T/Car ratios demonstrated light adaptation and absorption patterns, with a decrease in carotenoids (Car), leaving plants vulnerable to excess light exposure [41]. This could lead to chloroplast membrane peroxidation, chlorophyll degradation, and photosynthetic disruption due to metal interaction with -SH groups of biosynthesis-responsible enzymes [41,42,43]. However, AMF-colonized plants maintained relatively more stable pigment levels throughout the experiment, which may indicate an additional contribution of AMF to Cd detoxification.

Multiple studies have shown that AMF symbiosis can enhance antioxidant enzymatic activities, helping to reduce oxidative damage generated by ROS production when metabolic balance is altered by Cd presence [7,40,43]. Increased MDA levels likely resulted from Cd-induced damage to chloroplasts and electron transport chains, leading to protein decomposition through oxidative reactions [44]. Antioxidant enzymes play a crucial role in ROS elimination, minimizing cellular oxidation [40]. Specifically, SOD enzyme activation in the presence of AMF during initial hours suggests more effective superoxide radical (O_2_•^−^) elimination in sugarcane cells. This process catalyzes the conversion of superoxide radicals (O_2_•^−^) to H_2_O_2_, activating CAT, POD, and GPX enzymes, which convert H_2_O_2_ to H_2_O and O_2_, protecting various cellular compartments from oxidative damage [45,46].

The POD enzyme represents an alternative mechanism for H_2_O_2_ elimination, showing higher activation in response to this compound compared to CAT. However, this affinity can vary depending on the type of stress, whether biotic or abiotic [41]. In this study, the highest POD activity was observed in Cd treatment without AMF, indicating low enzyme affinity for AMF (Figure 4e). This behavior aligns with reports by Shi et al. [47] and Yang et al. [41], who demonstrated that POD response was not significant in *Carthamus tinctorius* L. exposed to Cd, nor in *Robinia pseudoacacia* L. with AMF under Pb stress. The CAT, an oxidoreductase present in peroxisomes and mitochondria, decomposes two H_2_O_2_ molecules [45,46]. Recent studies have shown that AMF stimulates CAT enzyme activity in plant leaves grown in Cd-contaminated soils [48,49], potentially generating more O_2_•^−^ dismutation reactions and H_2_O_2_ accumulation [50]. This H_2_O_2_ increase promotes CAT activation without requiring a reducing agent. However, when H_2_O_2_ levels decrease, its activity reduces, activating enzymes like APX that eliminate residual H_2_O_2_ using ascorbate as a reducing agent, thus protecting cells from oxidative damage [45,50], as observed at 120 days.

This behavior corresponds with Yousefi et al. [3] findings in sugarcane leaf samples exposed to Cd, where APX enzyme decreased as CAT response increased, demonstrating sugarcane’s high capacity to eliminate metal-induced ROS. Additionally, APX and GR enzymes participate in the ascorbate-glutathione (ASA-GSH) cycle, contributing to reactive oxygen species elimination in plants [51]. APX and GPX, present in the cytosol, cell wall, vacuole, and extracellular spaces [40], showed significant GPX activation in the early hours of sugarcane plants exposed only to Cd. According to Yang et al. [40], this is attributed to H_2_O_2_ catalysis and phenolic compounds for cell wall lignification processes, as well as possible GSH oxidation to GSSG under abiotic stress. This behavior might have reduced GSH and NP-SH concentrations, inhibiting GPX enzymatic activity after 48 h, and activating GR at 24 h to regulate the GSH/GSSG ratio [51]. The increase in APX at 120 days contributed to H_2_O_2_ reduction to water, using initial AA reserves as a reducing agent; GSH regenerates AA from its oxidized form DHA [51]. GSH is used by POD and GPX to reduce H_2_O_2_, oxidizing to GSSG; the transition back to the reduced form occurs through GR activation in the presence of GSSG and NADPH as a reducing agent [51,52].

GSH is essential for APX functioning and maintaining AA in its active form [50,51]. Additionally, in the presence of Cd and NP-SH, it promotes PC synthesis. The AMF contributed to GSH increase, generating metal tolerance over time (Figure 5a), induced by continuous Cd chelation through PC compound [3,7,51]. Therefore, AMF symbiosis helped sugarcane plants reduce oxidative stress and maintain active antioxidant enzyme response under Cd stress conditions (Figure 2). These results are achieved through metabolic pathways involving various antioxidant enzymes and electron donors. However, detailed information about the relationship between AMF and enzymes in sugarcane leaves remains limited, requiring further research. It is important to note that other antioxidant enzymes and/or non-enzymatic antioxidants might also be involved and should be considered for future studies. Although the presence of symbiosis with arbuscular mycorrhizal fungi (AMF) can modify the antioxidant defense systems of host plants, thereby reducing the proliferation of reactive oxygen species (ROS), the mechanisms involved may vary depending on the fungal species and the availability of micronutrients [26]. Several morphological studies have demonstrated a direct relationship with ROS, identifying the presence of H_2_O_2_ in the apoplastic space around the tips of the hyphae and in the final stages of the arbuscule’s life cycle, so the elimination of H_2_O_2_ is highly positively correlated with arbuscule richness [53].

Overall, multivariate analyses supported these findings, with the PCA showing a clear association of these enzymes with inoculated treatments. Pearson’s analysis confirmed these observations, revealing strong positive correlations between GSH, NP-SH, and the antioxidant enzymes CAT, APX, GR, GPX, and POD. These results highlight the integrative role of AMF in jointly regulating enzymatic activity, non-enzymatic antioxidants, and pigment dynamics, making a decisive contribution to stress mitigation.

## 4. Materials and Methods

### 4.1. Crop Establishment and Treatments

This study was conducted at the Center for Amazonian Research, CIMAZ Macagual César Augusto Estrada González, located in Florencia, Caquetá, Colombia (1°37′ N and 75°36′ W), an area characterized by a Tropical climate (AF) according to Köppen’s classification. For the experimental development, sugarcane (*Saccharum officinarum*) bud variety CC 01-1940 was used, provided by the CIMAZ-Macagual germplasm bank. The experimental substrate had been previously treated with a commercial phosphate-based fertilizer (special formula 2-4-6 + micronutrients) and was subsequently subjected to a disinfection process aimed at reducing the number of arbuscular mycorrhizal fungi. This process consisted of thermal treatment at 70–95 °C for 2 h, followed by exposure to solar radiation for 2 weeks [54]. Soil analyses revealed the following physicochemical characteristics: pH 4.74, organic matter 76.49 g kg^−1^, oxidizable carbon 33.69 g kg^−1^, phosphorus (P-Bray II) 86.35 mg kg^−1^, total bases 2.32 cmol kg^−1^, sulfur 70.55 mg kg^−1^, nickel 60.7 mg kg^−1^, and cadmium 0.16 mg kg^−1^.

The experimental design was implemented using 480 sugarcane plants, individually planted in 5 kg capacity bags. At planting time, 240 plants were inoculated with 500 g of commercial arbuscular mycorrhizal fungi (AMF) (SAFER MICORRIZAS M.A ^®^ to promote fungal colonization in the roots. After three months, four treatments were established under a completely randomized block design: a control treatment without AMF and without addition of CdCl_2_·H_2_O (S-F-Cd), a second treatment with AMF and 50 mg kg^−1^ CdCl_2_·H_2_O (S+F+Cd), a third treatment with AMF and without CdCl_2_·H_2_O (S+F-Cd), and a fourth treatment without AMF and with 50 mg kg^−1^ CdCl_2_·H_2_O (S-F+Cd). Each treatment had 40 experimental units, and the experiment was conducted under open field conditions, allowing for a realistic evaluation of the interactions between AMF and sugarcane plants under cadmium stress.

### 4.2. Determination of AMF Colonization and Cadmium Content Analysis

The assessment of arbuscular mycorrhizal colonization percentage was conducted three days prior to Cd treatment application, following the modified Phillips and Hayman method [55]. Root samples were thoroughly washed with distilled water and cut into 1 cm segments, then cleared by boiling in a KOH solution for 60 min. Samples were neutralized with HCl and stained with 0.05% trypan blue in lactoglycerol. Microscopic examination was performed at 10× and 40× magnification using a biological microscope. A “colonized field” was defined as a microscopic field in which at least one arbuscule, vesicle, or hyphal segment of arbuscular mycorrhizal fungi was present. Each field corresponded to the area visible within the eyepiece at the given magnification (0.18 mm^2^ at 40×). For each root sample, a minimum of 50 fields was randomly examined. The colonization percentage was calculated by dividing the number of colonized fields by the total observed fields and multiplying by 100 [56,57]. For cadmium content analysis, foliar and soil samples were collected both before and after the experiment (0 and 120 days). These samples were dried at 60 °C, ground, and sieved to 0.5 mm. Analysis was performed using Energy Dispersive X-ray Fluorescence Spectroscopy (HD XRF^®^) (Z-Spec E-max, East Greenbush, NY, USA), with cadmium concentration reported in mg kg^−1^.

### 4.3. Collection and Pretreatment of Samples for Biochemical Parameters

Foliar tissue sampling was conducted at multiple time points following Cd treatment application, starting at 7 a.m.: 0, 3, 6, 9, 12, 24, 48, 96 h, and at 15, 30, and 120 days. For each sampling event, three plants were randomly selected from each treatment group. The collected tissue samples were immediately macerated with liquid nitrogen. The macerated samples were stored at −80 °C in an ultra-freezer (Infrico Medcare, Lucena, Spain) until further analysis.

### 4.4. Analysis of Photosynthetic Pigments and Biochemical Stress Markers

Chlorophyll a, b, total chlorophyll, and carotenoids were determined following the method of Nishiyama et al. [58]. A sample of 0.5 g of macerated sugarcane leaves underwent three extractions with 80% acetone, followed by filtration. The absorbance was measured at 663, 642, and 470 nm using a Multiskan SkyHigh microplate reader (Thermo Fisher Scientific, Waltham, MA, USA) [59]. Malondialdehyde (MDA) content was determined by preparing an extract using 0.1 g of macerated tissue with 2 mL of 5% *w*/*v* trichloroacetic acid (TCA). The mixture was centrifuged at 4000 rpm at 25 °C for 10 min. The analysis was performed by combining 1 mL of the extract supernatant with 1 mL of 0.6% *w*/*v* thiobarbituric acid (TBA). This mixture was heated in a water bath at 98 °C for 10 min, and absorbance was measured at 532, 600, and 450 nm using a Multiskan SkyHigh microplate reader [60]. Proline content was analyzed following Bates’ method [61] with modifications. A 0.2 g sample of macerated sugarcane leaves was homogenized with 2 mL of 3% sulfosalicylic acid and centrifuged at 4000 rpm for 10 min; 1 mL of supernatant was combined with 1 mL of acetic acid-ninhydrin mixture and 1 mL of glacial acetic acid. The mixture was incubated at 98 °C for 30 min in a water bath, followed by extraction with 3 mL of toluene. Absorbance was measured at 520 nm using a Multiskan Go microplate reader (Thermo Fisher Scientific, Waltham, MA, USA) [62].

### 4.5. Enzymatic Antioxidant Defense System

Superoxide dismutase (SOD, EC 1.15.1.1) activity was measured based on its ability to inhibit the photochemical reduction of Nitroblue tetrazolium (NBT) following the Beauchamp and Fridovich method [63] with modifications. NBT photoreduction was monitored at 560 nm using a Multiskan SkyHigh microplate reader [64]. Catalase activity (CAT, EC 1.11.1.6) was determined by monitoring the decrease in absorbance at 240 nm using a Multiskan Go microplate reader, following Aebi’s method [65], which measures hydrogen peroxide (H_2_O_2_) consumption [66]. Ascorbate peroxidase activity (APX, EC 1.11.1.11) was measured according to the Ascorbate/peroxide method by Nakano and Asada [67], tracking the conversion of ascorbate to monodehydroascorbate and dehydroascorbate at 290 nm using a Multiskan Go microplate reader [68].

Glutathione reductase activity (GR, EC 1.8.1.7) was evaluated based on NADPH consumption during GSSG reduction, following the Foyer and Halliwal method [69] with modifications. The reduction was monitored at 340 nm using a Multiskan SkyHigh microplate reader [70]. Peroxidase activity (POD, EC 1.11.1.6) was determined using Kireyko et al.’s modified method [71], measuring the catalytic process of H_2_O_2_ with o-dianisidine, which forms a colored compound detectable at 436 nm using a Multiskan SkyHigh microplate reader [4]. Guaiacol peroxidase activity (GPX, EC 1.11.1.7) was determined using the Neves and Lourenço method [72] with modifications, measuring guaiacol oxidation in the presence of H_2_O_2_ at 470 nm using a Multiskan SkyHigh microplate reader [68]. All results were expressed per mg of protein content, determined according to Bradford’s method [73].

### 4.6. Non-Enzymatic Antioxidant Defense System

Phytochelatin (PC) concentrations were quantified by subtracting reduced glutathione (GSH) concentrations from non-protein thiols (NP-SH) [74]. An extract was prepared using 0.2 g of macerate and 2 mL of 15% *w*/*v* trichloroacetic acid (TCA), then centrifuged at 10,000 rpm at 4 °C for 10 min. Reduced glutathione (GSH) was determined using the Tawfik method [75], and non-protein thiols (NP-SH) were measured using Ellman’s method [76] with modifications. The production of NTB (2-nitro-5-thiobenzoic acid) was quantified due to the reduction of DTNB [5,5′-dithiobis-(2-nitrobenzoic acid)] at 412 nm using a Multiskan SkyHigh microplate reader. GSH was used as a standard for GSH measurements, and Cysteine was used as a standard for NP-SH measurements [77,78]

Ascorbic acid (AA) content was determined by preparing an extraction mixture of formic acid/methanol/water (1:24:25) with 0.1 g of macerate using ultrasound. The solution was filtered directly into vials using 0.22 µm filters (13 mm diameter) and analyzed by HPLC-UV (Shimadzu LC-2010A HT, Kyoto, Japan). Analysis was performed using a Luna C18 column (250 mm × 4.6 mm, 5 µm; Luna Phenomenex, Macclesfield, UK) with a binary mobile phase of water/formic acid (99:1, *v*/*v*) and acetonitrile, at wavelengths of 254 and 280 nm under isocratic gradient conditions, with a flow rate of 1 mL min^−1^. AA identification was based on chromatogram analysis and retention times. Ascorbic acid was used as a standard [79].

### 4.7. Statistical Analysis

A linear mixed model (LMM) was used to detect significant differences between the level of colonization, Cd content, and different variables of enzymatic and non-enzymatic antioxidant activity. Treatments were considered as fixed factors, and blocks as a random factor. The assumptions of normality and homogeneity of variance were verified through an exploratory analysis of residuals. Differences in the means of fixed factors were verified using the DGC test (*p* < 0.05). Additionally, principal component analyses (PCA) were performed with the different variables. The LMM was performed using the *lme* function from the *nlme* package. The PCA was performed using the *fviz_dend* and *fviz_pca_ind* functions, respectively, from the factoextra package. Statistical analyses were performed in the statistical software InfoStat version 2020 [80] and in the R language version 4.3.3 (R Development Core Team 2025) [81].

## 5. Conclusions

The study results demonstrated that arbuscular mycorrhizal fungi (AMF) established an effective symbiosis with sugarcane plants, achieving colonization rates of 64% in S+F-Cd and S+F+Cd treatments, significantly higher than the 35% observed in treatments without inoculum. This symbiosis resulted in effective cadmium retention in the roots, evidenced by a significant reduction in metal translocation to the leaves, where a concentration of 0.47 mg kg^−1^ was recorded in colonized plants (S+F+Cd) compared to 1.21 mg kg^−1^ in non-colonized plants (S-F+Cd). The presence of AMF demonstrated a protective effect against cadmium-induced oxidative stress. This was manifested in a significant decrease in lipid peroxidation (MDA) and proline content in AMF treatments. Although an initial reduction in photosynthetic capacity was observed, AMF-colonized plants maintained more stable levels of photosynthetic pigments throughout the experimental period, preserving Chl a/b and Chl T/Car ratios. The antioxidant defense system was enhanced by the presence of AMF through multiple mechanisms. Significant activation of key antioxidant enzymes was observed, including Catalase (CAT), Ascorbate Peroxidase (APX), Glutathione Reductase (GR), Superoxide Dismutase (SOD), and Peroxidase (POD). Particularly notable was the activation of SOD during initial hours in the presence of AMF, suggesting more effective superoxide radical elimination. Additionally, an increase in non-enzymatic antioxidant compounds was recorded, including Reduced Glutathione (GSH), non-protein thiols (NP-SH), and ascorbic acid (AA), with the highest concentrations of GSH and NP-SH observed at 3 and 24 h in S-F+Cd and S+F-Cd treatments. The AMF-sugarcane symbiosis proved to be an effective strategy for protection against cellular oxidative damage and reduction of cadmium toxicity. Principal component analysis explained 39.7% of the total data variability (Dim 1: 27.1%; Dim 2: 12.6%), confirming the significant correlation between AMF presence and activation of antioxidant defense systems. These results suggest that AMF application represents a viable biological strategy for phytoremediation and sustainable sugarcane cultivation in cadmium-contaminated soils, providing an ecological solution for crop management in areas affected by heavy metals.

## Figures and Tables

**Table 1:** Effect of AMF on Cadmium (Cd) concentrations (mg kg^−1^) in rhizosphere soil and sugarcane leaves over 120 days.

Treatments	Soil	Leaves
S-F+Cd	68.38 ± 4.55 ^A^	1.21 ± 0.10 ^A^
S+F+Cd	48.73 ± 4.55 ^B^	0.47 ± 0.10 ^B^
S-F-Cd	0.18 ± 4.55 ^C^	0.15 ± 0.10 ^B^
S+F-Cd	0.18 ± 4.55 ^C^	0.15 ± 0.10 ^B^

Data are represented as mean value ± S.E. Different letters indicate significant difference according to the DGC test at *p* < 0.05. Treatments S-F-Cd, S-F+Cd, S+F-Cd, S+F+Cd, (S: soil, F: Fungi; Cd: Cadmium 50 mg kg^−1^; +: Addition; -: Without addition).
